# Supplementary material for: MetaRibo-Seq measures translation in microbiomes
Source: Nat Commun. 2020 Jun 29;11:3268. doi: 10.1038/s41467-020-17081-z (PMC7324362; doi:10.1038/s41467-020-17081-z)
Supplement: Supplementary file 10 — Supplementary Data 7 [file 41467_2020_17081_MOESM10_ESM.zip › File2/Confidence_High_Taxonomy/222538_out.krona.html]

Javascript must be enabled to view this page.

members
magnitude
magnitudeUnassigned
count
unassigned
taxon
rank

222538\_out

6


SRS013687\_contig\_number\_contig-100\_5691.126404
1

superkingdom
2759
5

5
4751
kingdom

451864
subkingdom

SRS023914\_contig\_number\_18489SRS1041145\_contig\_number\_32966
5
2

phylum
3
4890

subphylum
3
147537

class
3
4891

order
4892
3

family
3
1156497

3
461281
genus

species

SRS051031\_contig\_number\_32589SRS053356\_contig\_number\_20004SRS098571\_contig\_number\_62477
36023
3
